# Reduction of Post-Harvest Injuries Caused by *Drosophila suzukii* in Some Cultivars of Sweet Cherries Using a High Carbon Dioxide Level and Cold Storage

**DOI:** 10.3390/insects12111009

**Published:** 2021-11-09

**Authors:** Manal Mostafa, Abir Ibn Amor, Naouel Admane, Gianfranco Anfora, Giovanni Bubici, Vincenzo Verrastro, Luciano Scarano, Maroun El Moujabber, Nuray Baser

**Affiliations:** 1 CIHEAM-IAMB—International Centre for Advanced Mediterranean Agronomic Studies, 70010 Bari, Italy; manal.mustafa93@gmail.com (M.M.); abirtunisie@gmail.com (A.I.A.); admane@iamb.it (N.A.); verrastro@iamb.it (V.V.); elmoujabber@iamb.It (M.E.M.); 2 Centre Agriculture Food Environment, University of Trento, 38098 San Michele all’Adige, Italy; Gianfranco.anfora@unitn.it; 3 Research and Innovation Centre, Fondazione Edmund Mach, 38098 San Michele all’Adige, Italy; 4 Consiglio Nazionale delle Ricerche, Istituto per la Protezione Sostenibile delle Piante, via Amendola 165/A, 70126 Bari, Italy; giovanninicola.bubici@cnr.it; 5 Scuola di Scienze Agrarie SAFE, Università degli Studi della Basilicata, 85100 Potenza, Italy; luciano.scarano@unibas.it

**Keywords:** spotted wing drosophila, post-harvest, sweet cherry

## Abstract

**Simple Summary:**

The spotted-wing *Drosophila suzukii* is an invasive fruit fly that causes high levels of damage to cherry fruits, which are, economically, a very important crop in the Puglia region. This pest infests mature fruits which are close to harvest, and when additional treatments are not allowed due to residue problems. The effect of infestation is a prolonged post-harvest time and up to 100% crop loss according to our field monitoring. The objective of our study was to find an effective post-harvest management of such infestations. We determined the growth rate of larval *D. suzukii* in infested fruits which were stored at low temperatures or treated with CO_2_ at low or room temperature; such post-harvest treatments were also tested in terms of the fruit quality at the end of the storage time, with respect to untreated infested fruits. When fruits were stored at low temperature in the presence of CO_2_ a consistent inhibition of larval growth was observed, with no apparent decrease of fruit quality parameters. After one month of storage, the quality parameters of treated, infested fruits were similar to the quality parameters of newly harvested fruits and non-infested fruits.

**Abstract:**

Efficient strategies are required in sweet cherry fruits to control the spotted wing drosophila (SWD), *Drosophila suzukii*, due to its adverse economic effect on farmers. Cold storage (CS) and storage with elevated carbon dioxide (CO_2_) are environmentally safe approaches for the pest control of stored fresh fruit. These strategies are effective in controlling a wide variety of insect species, without allowing toxic compounds to accumulate. The purpose of this study was to assess the effectiveness of a post-harvest application of CO_2_ treatment at 50%, cold treatment at 4 °C (CT), and a combination of both (CO_2_-CT) in controlling the early stages of SWD within four cultivars of freshly harvested cherry fruit, namely “Burlat-Bigarreau”, “Giorgia”, “Ferrovia”, and “Lapins”. In addition, an evaluation of the quality attributes of the cherries (skin firmness, berry firmness, strong soluble material, and titratable acidity) was carried out at harvest and after 10 and 20 days of storage. All treatments significantly reduced the rate of emergence of SWD when compared to the control (untreated cherry at 24 °C), and 100% SWD mortality was obtained in Burlat-Bigarreau (CO_2_-CT). In addition, over the entire storage time, the quality parameters were preserved in the samples stored at 4 °C and in the samples with combined treatments in comparison with the control.

## 1. Introduction

While the origins of *Drosophila suzukii,* the spotted wing drosophila (SWD), are in south-eastern Asia, it has spread throughout different areas of the world [1]. Even though drosophilids are widely known for their inability to fly for long distances, they can be spread quickly by regional winds, however, their main means of spreading and invasion is through the movement of goods and people [2,3,4,5]. Outside of its native habitat in Asia, *D. suzukii* was reported for the first time in the United States in 2008; European and Mediterranean countries (Spain, France, and Italy) identified it in 2009. Furthermore, *D. suzukii* has been detected in Asia, Europe, North and South America [6], and Africa [7]. There are still environmentally adequate areas with potential for *D. suzukii* occurrence in Oceania [6].

In some countries, such as Spain, Switzerland, and Austria, *D. suzukii* was found to be widespread. In other countries, this pest can be actionable under eradication, such as in Slovakia, where *D. suzukii* is transient [8]. SWD is a highly polyphagous, invasive pest that can be hosted by different economically important plant species. These hosts include blackberries, blueberries, cherries, peaches, raspberries, strawberries, grapes (wine and table), and different wild fruits. Apples, apricots, loquat, greenhouse mandarins, persimmons, and some other species are also affected by it [8,9,10]. Notably, a considerable limitation for pest management is due to its broad host range. This is important, not only because of the threat that *D. suzukii* poses to crops, but also because populations may survive in various places that could have different hosts, both cultivated and wild, which have varying ripening times throughout the year [4].

The direct impacts of *D. suzukii* on revenue amounts to little more than 800,000 Euros (EUR), i.e., about 2.6% of the estimated revenue potential [11]. A loss of 3 million Euros was observed from 2011 to 2013. Switzerland was similar to Italy regarding this assessed economic loss [10]. *D. suzukii* is considered a severe economic threat to soft summer fruits. The female in particular is a powerful economic threat, and in most cases it can be difficult to observe the infestation in the early stages, as the female lays eggs close to harvesting times, which are critical times to apply a specific treatment. Moreover, this timing allows eggs to develop to the larval stage, and larvae start to feed on the pulp, causing the apparent symptoms of deterioration after harvest [12].

Nevertheless, insecticides are restricted for conventional production because of the label constraints on the number of applications per season. Unfortunately, due to the lack of registered, effective bio-insecticides, organic producers are facing an even more difficult situation [13]. Therefore, in addition to post-harvest methods for organic farmers and producers, some field-level tactics, such as using cultural and IPM methods and introducing biological control agents (i.e., parasitoids, predators, and pathogens), have been adopted.

Some post-harvest methods and strategies against SWD have been proposed [14]. However, they are not efficient in preventing the post-harvest losses of sweet cherry due to this pest. Therefore, the objective of the present study was to investigate the effect of different post-harvest treatments of cherry fruits that are allowed for use on organic products. These treatments included low temperature (4 °C) and high carbon dioxide (CO_2_; 50%) application, for the control of *D. suzukii* injuries and the maintenance of infested cherry fruit quality at the same level as those products that were healthy. Notably, several previous studies regarded the efficacy of elevated CO_2_, which were created from using either a controlled atmosphere or modified atmosphere packaging, against several pests, including *D. suzukii* [15,16,17,18]. Instead, the proposed methodology is composed of the application of 50% CO_2_ for 24 h in combination with cold storage.

## 2. Materials and Methods

*D. suzukii* adults were collected from the experimental field of CIHEAM-Bari (Valenzano, Puglia, Italy) in 2012. Periodically, fresh populations of the pest that were collected from infested fruits were added to rearing populations, to avoid problems associated with multi-annual breeding. Moreover, as a preventive method, all cages were regularly checked to avoid contamination from other *Drosophila* spp. which were reared in insectarium facilities of CIHEAM-Bari.

The insect rearing took place within growing rooms in controlled conditions (22 ± 2°C, 62 ± 4%, 14 h light/10 h dark). *D. suzukii* adults were reared in Plexiglass cages (50*40*40 cm), that allowed ventilation, and were fed on a cornmeal diet medium; water was supplied every 2–3 days. Feed was prepared by mixing 58.8 g corn flour, 76.5 g yeast, 58.8 g sugar, 2.1 g methyl-4-hydroxybenzoate (PMB) and 2.1 g agar–agar in 1 L of boiling, distilled water. The resulting suspension was continuously mixed every 2 min and boiled for 30 min. Afterwards, the suspension was cooled to 25 °C, and 2.5 g mixed vitamins and 1.8 mL propionic acid (99%) were added. Finally, the suspension was kept in vials at 4 °C [19].

The experiment was carried out using four cultivars of organic cherry: “Burlat-Bigarreau”, “Giorgia”, “Ferrovia”, and “Lapins”. These cultivars were planted and harvested from a commercial organic farm called ‘Tenute D’Onghia’, (Lat. 40,80236; Long. 16,86327), located west of the municipality of Gioia del Colle, in Taranto province, Southern Italy. The same amounts of cherries were randomly collected from each cultivar.

Before the application of the treatments that commenced the experiments, the estimation of the life cycle of *D. suzukii* inside the cherries, under the available conditions in the insectarium, was carried out. Twenty cherry fruits were placed in a cage in which *D. suzukii* was present for artificial infestation. After 1–2 days, the infested fruits were moved to 24 °C and monitored. To monitor the development of *D. suzukii*, each fruit was cut, and the development of their different stages was observed daily.

The harvested cherries were transported directly to the CIHEAM-Bari insectarium, where a simulation of natural infestation was performed. Fruits were chosen randomly, and carefully checked under a stereoscope (Nikon SMZ 745T) to exclude damaged and unhealthy berries. Healthy berries were added to the rearing cage to allow *D. suzukii* adults to lay eggs on them. After 1–2 days, which was the ovipositional period that was determined by the life cycle estimation, the infested fruits were removed from the cage, and the number of eggs per berry was counted under the stereoscope.

Infested samples from each cultivar were subjected to the following different treatments: (1) 50% CO_2_ at room temperature (24 °C); (2) cold treatment at 4 °C; (3) 50% CO_2_ at 4 °C; (4) the infested samples that were not treated with CO_2_ and were maintained at 24 °C were used as controls. The application of the CO_2_ treatment was carried out at Basilicata University using a semi-automatic, vacuum thermo-sealing machine that was used in operation with a modified atmosphere (Model UNICA 20, VALKO, Italy). The values of adult emergence were obtained as the means of 4 replicates per treatment, considering that 10 berries were used in each replicate. For each replicate, the numbers of eggs, berries showing insect emergence, and emergent adults were counted daily.

The mechanical and chemical quality parameters of the fruit samples were measured immediately after harvest, before artificial infestation, and after infestation at each sampling time (after 10 and 20 days of storage).

Regarding a fruit’s physical characteristics, a fruit’s firmness is often used as a measure of quality [20]. Physical characteristics. expressed in Newtons (N), were measured using a penetrometer (Digital Fruit firmness tester, TR Turoni, Italy). These attributes included the maximum force necessary to puncture the skin of an individual fruit with a 2 mm probe that penetrated to a depth of 6 mm (skin firmness), and the force required to compress a fruit through a flat cylinder probe with an 8 mm diameter to reach a depth of 5 mm (berry firmness).

Regarding the chemical analysis, the fruits were filtered through a plastic bag (BagPage^®^) which was fitted with a filter to extract the juice. The soluble solid content (SSC-Brix%) was determined with a digital refractometer (Atago, Japan), and the titratable acidity (TA), expressed in percentage of malic acid, was measured using a titration with 0.1 N NaOH up to pH 8.1 [21]. Finally, the pH of the juice was quantified by a pH meter (Crison, Spain).

The statistical analysis was carried out with R, version 3.6.5 (https://cran.r-project.org, last accessed date 31 August 2019), within RStudio, version 1.2.5042 (https://rstudio.com, last accessed date 31 August 2019). The percentage of emergence, as well as the number of fruits with emergence, were analysed using binomial models (*binomial* family within the *glm* function). The number of eggs, larvae, pupae, and adults were analysed using negative binomial models, with the *glm.nb* function of the package MASS v7.3-51.4. The variables of the cultivar, CO_2_ treatment, and temperature were used as fixed factors. Since the interactions between the main factors occurred for several parameters (e.g., percentage of emergence and the number of fruits with emergence), pairwise comparisons of means were performed for the simple effects. They were performed by contrasts after the *glm* or *glm.nb* functions, or by the Tukey’s HSD test after the *manova* function. Results of the contrasts were manually converted into significance letters to be reported in the graphs for better readability.

## 3. Results

### 3.1. Emergence Rate

The obtained results revealed that there was a non-normal distribution, due to major variations in the number of eggs per fruit in each cultivar. Additionally, the numbers of eggs per 40 fruits, per cultivar, were not standardized; Burlat-Bigarreau had the lowest number, with 745 eggs, while Giorgia, Ferrovia, and Lapins contained 2107, 3317, and 1253 eggs, respectively. As shown in Table 1, the number of eggs was influenced mostly by the cultivar.

**Figure 1:**
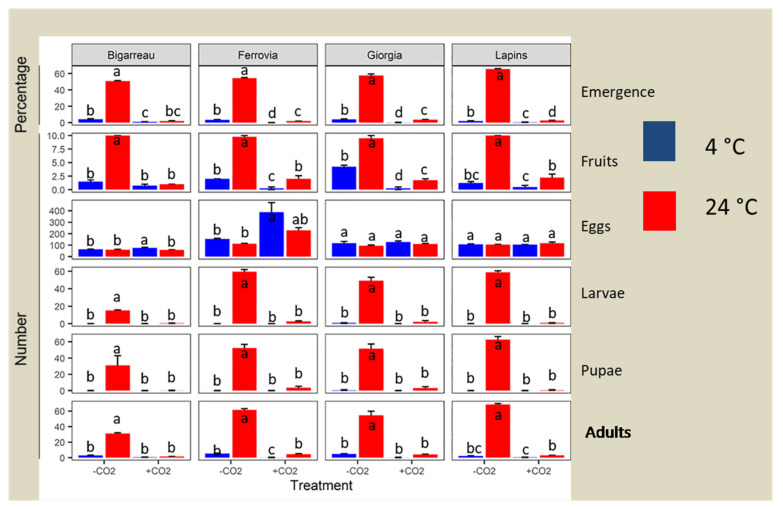
Several measurements of the insects, recorded on four cherry cultivars, treated or not with CO2, at 4 or 24 °C. Within each graph, columns with different letters are significantly different according to contrasts (*p* < 0.05). The number of fruits with emergence was analysed using a binomial model, while the number of eggs, larvae, pupae, and adults were analysed using negative binomial models.

All treatments were effective in reducing the emergence rate of the insects (Table 1); some replicates showed a 100% mortality rate. CO_2_ + 4 °C and CO_2_ + 24 °C had the same significance values, with *p* values ˂0.001. A slight difference was observed between the treatments used; however, the effects of the CO_2_ + 4 °C and CO_2_ + 24 °C treatments did not differ significantly from the effect of the 4 °C treatment (Figure 1).

Regarding the mortality rate, the exposure period for 100% mortality of *D. suzukii* in the cherry fruits was 48 h following CO_2_ application at 4 °C, whereas 4 and 8 days were needed for eggs and larvae, respectively, to achieve 100% mortality under 4 °C treatment in all cultivars except Giorgia, where few pupae were observed.

Moreover, all treatments (4 °C, CO_2_ + 24 °C, and CO_2_ + 4 °C) were more efficient on a smaller number of fruits in all cultivars and led to less emergence than seen in the negative controls (24 °C). Nevertheless, the CO_2_ + 4 °C treatment showed a significant reduction in three cultivars, namely Ferrovia, Giorgia, and Lapins (Figure 1).

Few pupae were observed in Giorgia fruits, as well as in the CO_2_ + 4 °C treatment for all cultivars. Moreover, in comparison with the negative control (24 °C), all treatments (4 °C, CO_2_ + 24 °C and CO_2_ + 4 °C) had the highest effect with a smaller number of fruits, however emergence was observed in all cultivars. Nevertheless, the CO2 + 4 °C treatment showed a significant reduction in three cultivars, namely Ferrovia, Giorgia, and Lapins (Figure 1).

The preliminary analysis indicated that all treatments induced a reduction of the larval and pupal development of *D. suzukii* when compared to the negative controls (24 °C). Furthermore, variations in the number of emerged individuals were observed between the treatments, temperatures, and cultivars used (Table 1). However, the elaboration of the data with negative binomial models showed that the impact of this reduction was only influenced by the treatment effect (Figure 1). Nonetheless, the number of larvae in the study included both live and deceased larvae that were found in treated fruits, except for the controls.

### 3.2. Quality Parameters

**Figure 2:**
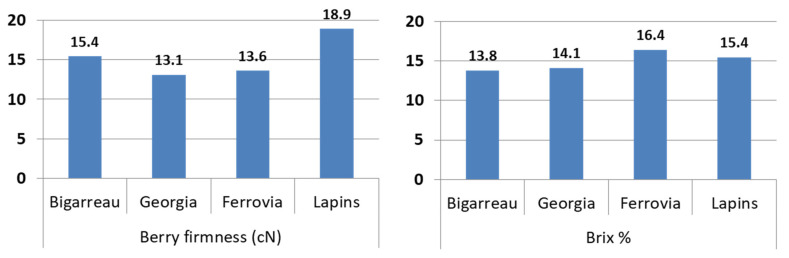
Berry firmness and Brix % of four cherry cultivars, Bigarreau, Giorgia, Ferrovia, and Lapins; berry firmness is expressed as Newton Units and Brix as a percentage.

The berry firmness and % Brix of the four tested cherry cultivars were measured before artificial inoculation was performed (Figure 2). Lapins had the highest berry firmness value (18.9 cN), while Ferrovia had the highest Brix value (16.4 %). Additional quality parameters, such as the skin firmness and titratable acidity (TA), were determined at inoculation and at both 10 and 20 days after inoculation. Significant changes in these 4 parameters were determined due to both the different cultivars and treatments used, namely with CO_2_ at room temperature or CO_2_ at 4 °C (Table 2). Moreover, it was determined that time (0, 10, 20 days after inoculation) was an effective variable of significant changes among quality parameters. The analysis of variance showed that there were no significant differences in berry and skin firmness due to the treatments, while all parameters were influenced by the cultivar and time interaction (Table 2). Over 20 days, the four parameters of cherry fruits treated with CO_2_, with or without refrigeration, showed analogous changes over time; significant changes were found in the different cultivars.

**Figure 3:**
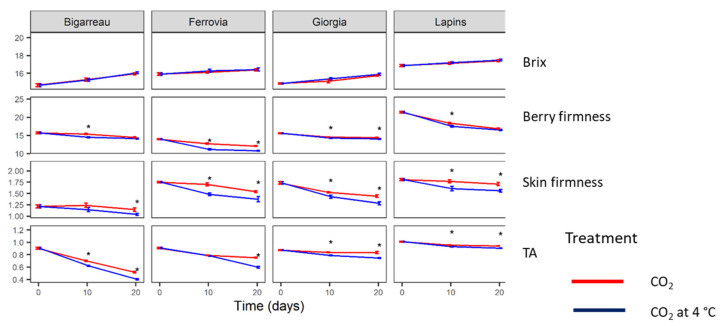
Brix (%), berry firmness (cN), skin firmness (cN), and TA (titratable acidity, g/L) measured in the fruits of four cherry cultivars that were treated with CO_2_ with or without refrigeration (4 °C). Asterisks indicate significant difference between treatments, according to the Tukey’s HSD test (*p* < 0.05).

As shown in Figure 3, the parameters were reduced over time except for SSC, which is represented by Brix.

## 4. Discussion

The fruits of the Burlat-Bigarreau cultivar tended to have the lowest firmness when compared to the other cultivars [22]. However, it also had the lowest number of eggs (745 eggs) when compared to the other cultivars, namely Ferrovia (2107 eggs), Giorgia (3317 eggs), and Lapins (1253 eggs).

Furthermore, according to unpublished data, Burlat-Bigarreau showed a low infestation rate of *D. suzukii* and cherry fruit fly (*Rhagoletis cerasi*) during several years of monitoring in the field [23].The main reason is that Burlat-Bigarreau is an early variety which has a low protein to carbohydrate (P:C) ratio [24] when compared to the substrate in the cage during artificial infestation, when *D. suzukii* is in the post-overwintering period [25].

In this study, this could be the cause of the lower SSC observed in Burlat-Bigarreau during the experiment when compared to the substrate and the other three varieties, which had a higher SSC, and which represents their carbohydrate content (Figure 2). *D. suzukii* prefers to oviposit in fruits with a low protein content, whereas carbohydrate content tends to be high [25,26]. In addition, many studies have demonstrated a positive correlation between *D. suzukii* oviposition rate and SSC [27,28,29]. Since Ferrovia had the highest Brix value, as shown in Figure 2, which indicates a high carbohydrate content when compared to Burlat-Bigarreau, consequently, Ferrovia had the highest egg number.

Many studies have been conducted to evaluate the efficacy of post-harvest cold treatments on *D. suzukii* development. For instance, Aly et al. [30] and Kim et al. [31] studied the effect of cold treatment on *D. suzukii,* which indicated that cold treatment reduced individuals’ survival and increased the larval development duration. Similarly, our results support this prior study, as the immature individuals’ development duration increased with the cold treatments when compared to the 24 °C treatments. However, in the cherry fruits used in the cold treatments, development did not continue to the pupal stage. Additionally, the survival rate was relatively high, and the emergence rate was significantly lower.

The effect of CO_2_ was investigated in different studies for the control of various post-harvest pests [16,17,18,32,33]. Regarding the control of *D. suzukii,* Follett et al. [15] studied the effect of low oxygen (elevated CO_2_) on individuals’ development and its effect on their resistance to irradiation, a post-harvest treatment. All previous studies demonstrated an effect of CO_2_ that was created either from a controlled atmosphere or from modified atmosphere packaging. Alternatively, our methodology depended on the use of CO_2_ at a constant percentage of 50% for 24 h.

Our results confirmed the effectiveness of all treatments in reducing the number of the hatching eggs, larvae, and pupae of SWD, thus reducing the number of fruits with emergence. There were no significant differences among treatments.

Furthermore, the greatest number of individuals was recorded for the cultivar Lapins, with the other cultivars showing similar values. Notably, the Ferrovia cultivar in the CO_2_ + 4 °C treatment showed a significant reduction in the emergence rate (*p* ˂ 0.05). However, this significance may not have resulted from the treatment effectiveness, but by the negative correlation between the high number of eggs and the emergence rate [34]. Furthermore, this was confirmed by Wang et al. [35], which indicated that the emergence rate was reduced when the number of eggs increased per gram of density in fruits. Moreover, the more eggs present in one fruit, the lower the emergence of adults, probably due to the increased competition amongst larvae for food and space.

Considering the number of pupae, the results were the same as for the number of individuals; thus, there was a significant reduction of pupae in all treatments when compared to the control, and no differences were observed among treatments.

In addition, Lapins had the greatest number of pupae when compared to the other cultivars, among which no differences were observed.

Overall, low temperatures alone were effective for reducing the risk of eggs hatching during cherry storage. The addition of CO_2_ had no significant benefits when compared to the low temperature (4 °C) alone; thus, our results indicate that SWD in post-harvest can be efficiently controlled by the storage of fruits at low temperatures, without the need for further energetic, and costly, inputs of CO_2_. However, the application of CO_2_ was effective in regard to the mortality time when compared to the low temperature (4 °C) alone.

Moreover, our results suggest that cultivars, such as Lapins, provide a better environment for SWD development and shelter the insects from unsuitable external conditions more than other cultivars, namely Ferrovia, Giorgia and Bigarreau. This may be due to the high firmness of the Lapins cultivar during the experiment time (Figure. 2). According to Aly et al. [30] fruits that are thicker and harder than other cultivars may provide a place for individuals to seek shelter, and could explain the high emergence rate in the Lapins cultivar when compared to the other cultivars.

Regarding the quality parameters, throughout ten days of storage at 24 °C, all the fruits were lost due to either fungal decay or the natural decay of the fruits. Consequently, the fruits lost their marketability value and were excluded from the trial after the first sampling time (10 days of CS). When compared to the initial values that were obtained at harvest time, the physical and chemical parameters did not vary significantly among both treated samples at the different sampling times during the CS period.

Regarding the samples stored at a low temperature of 4 °C and with combined treatments (CO_2_ + 4 °C), no significant differences were registered in the tested quality parameters, namely the berry firmness, skin firmness, SSC, and TA. Moreover, all of these attributes were influenced by the cultivar throughout the storage period.

Many studies have indicated that the storage temperature does not correlate with cherry firmness but with the cultivar [34,35,36], which confirms our results. However, differences between the firmness of the cultivars were observed, which could be due to variations in skin features, cell structures, and composition rate [36]. Some studies found an increase in berry firmness with cold storage [37,38] and a high level of CO_2_ that was generated by post-harvest technology, such as modified atmosphere packaging (MAP) [15,16,17,18,39,40]. In addition, late season cultivars are often firmer than early season cultivars [41], which may explain the high value of firmness for the Lapins cultivar when compared to Burlat-Bigarreau, which is an early season cultivar.

Esti et al. [42] reported that storage at low temperatures of 0 and 1 °C influenced SSC and TA. However, in our experiment, no significant differences were observed between the two treatments in all cultivars during the storage period. SSC and TA increased during the storage period due to water loss, likely because cherries are highly perishable fruits [40,43]. Furthermore, the differences between cultivars could also be due to environmental conditions and pre-harvest treatments [43]. Nevertheless, it is crucial to highlight that no study exists regarding the maintenance of quality for infested fruits, as in the case of our experiment.

## 5. Conclusions

The results of the current study indicated that the storing of infested cherry cultivars (Burlat-Bigarreau, Giorgia, Ferrovia, and Lapins) at 4 °C for eight days controlled SWD, while 24 h exposure to 50% CO_2_, in combination with cold storage, controlled all stages of SWD after 48 h. However, in the only study regarding the control of SWD in sweet cherries, CO_2_ was created using MAP, which is expensive when compared to simple exposure to CO_2_ for 24 h.

According to our findings, it is safe to recommend that farmers use a combination treatment that consists of 24 h exposure to CO_2_ at 4 °C for 48 h. Due to its effectiveness, achieving a 100% mortality rate for *D. suzukii* within 48 h, a lower cost when compared to other post-harvest treatments, and its safeness for organic production, this treatment is recommended.

In conclusion, the obtained results represent the first step in defining a large-scale post-harvest protocol (packing house) to allow sweet cherry producers to sell their field-infested fruits as healthy and fresh, without any toxic residues that are risky for consumers.

Further research is required to validate the proposed protocol on other cultivars and stone fruits and to understand the mechanism of treatment on both SWD mortality and the quality attributes of the fruit.

## Figures and Tables

**Table 1:** Analysis of variance for several measurements on the insects.

Source of Variability	*P > LR*					
	E	F	Eg	L	P	I
Cultivar	0.012	0.691	<0.001	<0.001	<0.001	<0.001
CO_2_ Treatment	<0.001	<0.001	<0.001	<0.001	<0.001	<0.001
Temperature	<0.001	<0.001	<0.001	<0.001	<0.001	<0.001
Cultivar × CO_2_ Treatment	0.05	0.226	<0.001	0.172	0.01	<0.001
Cultivar × Temperature	<0.001	0.417	<0.001	0.051	0.057	<0.001
CO_2_ Treatment × Temperature	<0.001	<0.001	0.489	0.606	0.563	<0.001
Cultivar × CO_2_ Treatment × Temperature	<0.001	0.019	0.190	1	1	<0.001

E = percentage of emergence; F = number of fruits with emergence; Eg = number of eggs per 10 fruits; L = number of larvae per fruit; P = number of pupae per fruit; I = number of individuals per 10 fruits.

**Table 2:** Repeated measure (0, 10, and 20 days) analysis of variance for Brix, berry firmness, skin firmness, and TA.

Source of Variability		*P > F*		
	Brix	Berry Firmness	Skin Firmness	TA
*Between subjects*				
Cultivar	<0.001	<0.001	<0.001	<0.001
Treatment	<0.001	0.044	0.041	<0.001
Cultivar × Treatment	0.943	0.015	0.752	<0.001
				
*Within subjects*				
Time	0.006	<0.001	<0.001	<0.001
Time × Cultivar	<0.001	<0.001	<0.001	<0.001
Time × Treatment	<0.001	<0.001	<0.001	<0.001
Time × Cultivar × Treatment	0.894	0.76	0.713	0.048

## Data Availability

Data is contained within the article.
